# Intraoperative Isoflurane End-Tidal Concentration during Infusion of Fentanyl, Tramadol, or Fentanyl–Tramadol Combination in Cats

**DOI:** 10.3390/vetsci11030125

**Published:** 2024-03-11

**Authors:** Claudia Interlandi, Fabio Bruno, Marco Tabbì, Francesco Macrì, Simona Di Pietro, Elisabetta Giudice, Patrizia Licata, Daniele Macrì, Viola Zappone, Giovanna Lucrezia Costa

**Affiliations:** 1 Department of Veterinary Sciences, University of Messina, Via Palatucci Annunziata, 98100 Messina, Italy; cinterlandi@unime.it (C.I.); fabio.bruno@unime.it (F.B.); francesco.macri@unime.it (F.M.); simona.dipietro@unime.it (S.D.P.); elisabetta.giudice@unime.it (E.G.); patrizia.licata@unime.it (P.L.); viola.zappone@unime.it (V.Z.); glcosta@unime.it (G.L.C.); 2 Zooprophylactic Institute, Via Gino Marinuzzi 3, 90129 Palermo, Italy; daniele.macri@izssicilia.it

**Keywords:** isoflurane, fentanyl, tramadol, slow infusion, analgesia, cats

## Abstract

**Simple Summary:**

The study evaluated the intraoperative isoflurane end-tidal concentration (isoflurane-sparing effect), clinical parameters, intraoperative antinociceptive effect, and postoperative analgesia in cats undergoing ovariohysterectomy treated with fentanyl, tramadol, or a combination of both. A reduction in the end-tidal isoflurane fraction and better analgesia were observed during fentanyl/tramadol infusion while no differences were found between the two drugs used alone. The results of this study suggest that the combination of fentanyl and tramadol could be proposed as a viable alternative anesthetic protocol in cats undergoing ovariohysterectomy.

**Abstract:**

The aim of this study was to evaluate the end-tidal concentration of isoflurane required, clinical parameters, intraoperative antinociceptive effect, and postoperative analgesia in cats undergoing ovariohysterectomy, receiving fentanyl, tramadol, or fentanyl/tramadol. Sixty-six cats in three groups, were premedicated with dexmedetomidine and infused with one of the following treatments: fentanyl, tramadol, or fentanyl/tramadol combination. Anesthesia was induced with alfaxolone and maintained with isoflurane, titrated to keep heart rate, respiratory rate and systolic arterial pressure within target values recorded at endotracheal intubation. An intraoperative cumulative scale was performed. Postoperatively, a short form of the Glasgow Composite Measure Pain Scale Feline was used at 2, 12, and 24 h. The groups were similar for age, weight, dose of dexmedetomidine, and alfaxalone administered. A greater reduction in the end-tidal isoflurane fraction was observed with the combined fentanyl/tramadol infusion than with either fentanyl or tramadol alone. No differences in the end-tidal isoflurane fraction were found between fentanyl or tramadol alone. Hemodynamic stability associated with minimal cardiopulmonary changes, low response to noxious intraoperative stimulation, and low postoperative pain scores were also observed with the fentanyl/tramadol combination. The fentanyl/tramadol combination provided a reduction in the end-tidal isoflurane fraction compared with fentanyl or tramadol alone.

## 1. Introduction

Multimodal anesthesia combines different drugs to reduce their doses and toxic effects [1]. This technique can be very useful in feline patients, where the choice of analgesics and their dosage must consider the lack of many drug conjugation and metabolism pathways in this species, resulting in prolonged plasma concentrations, deficiencies in hepatic metabolism (decreased UGTC), and decreased excretion, with increased toxicities and other side effects [2,3,4]. While the reduction of inhaled anesthetic requirements by opioid administration has been demonstrated in dogs, conflicting data have been reported in cats [1,5,6,7,8,9,10,11,12,13,14,15,16]. Fentanyl is a potent µ-opioid receptor agonist used in cats [15,17,18]. However, the distribution of opioid receptors in the feline central nervous system remains unclear [4]. It has been reported that the infusion rate of 3 to 5 μg/kg/h of fentanyl provides a rapid onset and good analgesic effect during the infusion period, which is rapidly lost when the infusion is stopped [19,20]. Tramadol and its active metabolite (M1 O-desmethyltramadol), bind to µ-opioid receptors in the central nervous system, activating the monoaminergic descending inhibitory pathway to the spinal cord and reducing noradrenalin and serotonin reuptake into the neuronal terminal responsible for pain relief [6,10,20,21]. Tramadol is eliminated more slowly in cats than in dogs [7,22,23,24,25,26,27]. Some authors have reported that this analgesic causes several dose-related adverse effects, which are significantly more frequent at a dose of 4 mg/kg than at 1 mg/kg [28,29,30]. The present study was designed to compare the effects of fentanyl, tramadol, or a combination of both on the concentration of isoflurane, clinical parameters, and assessment of analgesia in cats undergoing ovariohysterectomy.

## 2. Material and Methods

### 2.1. Animals

The protocol of animal husbandry and experimentation was reviewed and approved in accordance with the standards recommended by the Guide for the Care and Use of Laboratory Animals and Directive 2010/63/EU for animal experiments. This study was approved by the Ethical Committee of the Department of Veterinary Sciences, University of Messina (approval no.: 074/2022).

Sixty-six (*n* = 66) female domestic short-hair cats with a mean age of 2.3 ± 1 years (mean/SD) and a mean body weight of 3 ± 2 kg (mean/SD) undergoing ovariohysterectomy for birth control, were included in the study. Written informed consent was obtained from all owners prior to participation in the study. All cats were examined prior to pre-medication. Only cats in the American Society of Anesthesiologists (ASA) category I were included in the study based on the results of physical examination, complete blood count, and serum biochemistry. Hematological and biochemical tests (packed cell volume, total protein, albumin, alanine transaminase, aspartate transaminase, alkaline phosphatase, gamma-glutamyl transferase, total bilirubin, creatinine, urea nitrogen, glucose, and pH) were within the reference ranges. Cats that were not in ASA category I, allergic to opiates, and had been treated with analgesics or anti-inflammatories within the previous 7 days were excluded from the study. Cats whose owners had requested laparoscopic surgery were also excluded. Cats were fasted overnight with free access to water for up to 2 h before being admitted to the hospital. Surgery was performed the same morning. Cats were randomly assigned by lottery to one of three treatment groups: fentanyl group (F Group; *n* = 22), tramadol group (T Group; *n* = 22), and fentanyl/tramadol group (FT Group; *n* = 22).

### 2.2. Treatments

All subjects were premedicated with dexmedetomidine 5 µg/kg (Dexdomitor^®^, 0.5 mg/mL Vetoquinol Italia SRL, Bertinoro, Italy), injected intramuscularly (IM) into the femoral biceps. After premedication, subjects were maintained in a quiet environment for 10 min to observe behavioral drug effects, such as changes in locomotor activity, posture, and achievement of sternal or lateral recumbency. A 24G (0.74 mm) intravenous catheter was aseptically inserted into both cephalic veins for intravenous (IV) drug administration. Anesthesia was induced with intravenous alfaxalone 3 mg/kg (Alfaxan 10 mg/mL Dechra Veterinary Products SAS, FR), over approximately 60 s, titrating the dose according to the patient’s response. The trachea was then intubated with a 3 or 3.5 ID endotracheal tube connected to an anesthetic machine with an isoflurane vaporizer (GE Datex-Ohmeda Avance 6.0; Helsinki, Finland). Anesthesia was maintained with isoflurane in 100% oxygen (Vetflurane 1000 mg/g Virbac S.r.l., Milano, Italy) administered via a T-piece breathing system. The oxygen flow rate was maintained at 200 mL/kg/min. All animals were allowed to breathe spontaneously. The vaporizer was initially set to 2% isoflurane. If HR and RR were within the reference range for anesthetized cats [31], the Fe’ISO was reduced, but never below 0.8%. A 20% increase in either HR and/or RR or increased jaw tone, strong palpebral reflex or central rotation of the eyeball, and spontaneous movements were considered to indicate inadequate anesthesia. Intravenous opioids were administered as a 30-s bolus after intubation in all groups, followed by CRI. In the FT group, fentanyl and tramadol were administered simultaneously via two separate venous accesses. In groups F and T, 0.9% saline (0.9% sodium chloride; S.A.L.F. S.p.A. Bergamo, Italy) was administered during CRI via the venous access contralateral used for opioid administration to avoid observer bias due to the absence of a second infusion pump. Surgery was started 10 min after the start of CRI. CRI was discontinued after the administration of halogenated solution was stopped. Group F received a bolus of fentanyl at 1 µg/kg (Fentanest 50 µg/mL Pfizer Italia S.r.l., Milano, Italy) followed by CRI at 5 µg/kg/h. The T group received a bolus of tramadol at 1.5 mg/kg (Altadol 50 mg/mL Formevet S.r.l., Milano, Italy) followed by CRI at 2.6 mg/kg/h. The FT group received a bolus of fentanyl at 0.5 µg/kg followed by CRI at 2.5 µg/kg/h and a bolus of tramadol at 0.8 mg/kg followed by CRI at 1.3 mg/kg/h. For the CRI infusion, the analgesics were diluted in 20 mL of saline and administered using an infusion pump (Perfusor^®^ compact ^plus^, B. Braun Milano (S.P.A.), Milano, Italy). In the operating theatre, cats were placed in dorsal recumbency, and the temperature was maintained at 37.0 °C to 38.5 °C with a circulating warm-water blanket (PlastiPad^®^, Gentherm, Sharonville, OH, USA). Clinical functions were assessed using a multiparameter monitor (Datex-Ohmeda S/5; Helsinki, Finland), which continuously displayed heart rate (HR, beats/min), non-invasive systolic (SAP), mean (MAP), and diastolic (DAP) arterial blood pressure (mmHg), hemoglobin oxygen saturation (SpO_2_, %) and esophageal temperature (T, °C). A pulse oximeter was placed on the tongue to record pulse rate (HR) and arterial hemoglobin oxygen saturation (SPO_2_). A pediatric cuff, approximately 40% of the circumference of the leg, was placed just below the elbow to non-invasively record systolic (SAP), mean (MAP), and diastolic (DAP) blood pressures using the oscillometric method. Respiratory gas was continuously sampled by a side-stream capnograph with a low dead space in-line endotracheal tube connector inserted between the breathing circuit and the endotracheal tube. Respiratory rate (RR, breaths/min), end-tidal CO_2_ (EtCO_2_, mmHg), and end-tidal fraction of isoflurane (Fe’ISO) were measured by the capnograph and displayed on the respiratory monitor of the anesthesia machine (GE Datex-Ohmeda Avance 6.0; Helsinki, Finland). Changes in these parameters during anesthesia were recorded and analyzed. The analyzer was calibrated at the beginning of the experiment with the calibration gas provided by the manufacturer (Quick Calibration Gas, Ref: 755583; GE Healthcare, Chicago, IL, USA). A thermistor, calibrated daily against a certified thermometer, was inserted into the thoracic portion of the esophagus to monitor core body temperature (°C) (Datex-Ohmeda S/5). All surgeries were performed by a single experienced veterinary surgeon who was blinded to the treatment group. Ovariohysterectomy was performed via a standard ventral midline approach. All patients received intravenous meloxicam at 0.1 mg/kg (Metacam 0.5 mg/mL Boehringer Ingelheim Italia S.p.A, Milano, Italy) ten minutes after discontinuation of CRI. Cardiovascular (HR, SAP, MAP and DAP) and RR data were collected before premedication, during endotracheal intubation (T1), skin incision (T2), right ovarian pedicle traction (T3), left ovarian pedicle traction (T4), ligation of the uterine body (T5), initiation of peritoneal suturing (T6), and skin suturing (T7) to assess nociceptive responses to surgical stimulation and the efficacy of opioid treatment. Gas exchange (EtCO_2_, SpO_2_, Fe’ISO) and T° data were collected at the same intraoperative time points but from endotracheal intubation (T1). A cumulative numerical scale reporting the response to intraoperative noxious stimulation, modified from previous studies [32,33,34,35,36], was used to score the percentage changes in HR, RR, and SAP compared with basal values (T1) according to the following procedure: (time point value − basal value)/basal value × 100 = % change. Values recorded during endotracheal intubation were considered as basal values (T1). The scoring of the variables was determined according to the following criteria: Score 0 ≤ 0%: basal value did not change or decreased; Score 1 > 0% but ≤10%: basal value increased up to 10%; Score 2 > 10% but ≤20%: basal value increased from 11% to 20%; Score 3 > 20% but ≤30%: basal value increased from 21% to 30%; Score 4 > 30%: basal value increased more than 30%. All scoring was conducted by the same evaluator. The sum of the scores assigned to the selected variables provided the total score, which ranged from a minimum of 0 to a maximum of 12. If HR, RR, and SAP increased by more than 20% to a score of 6 or higher, rescue analgesia was administered with a 2 µg/kg fentanyl bolus. Subjects who required rescue analgesia were to be excluded from the study. However, no subjects required rescue analgesia and hence no subject was excluded from the study. Anesthesia was performed by a single anesthetist. Throughout the study, all analyzed parameters were assessed by 3 independent observers. The observers were blinded to the treatment administered. Extubation time was calculated from the time the vaporizer was switched off until the appearance of the laryngeal reflex. The time to reach the sternal position was recorded after extubation. A short form of the Glasgow Feline Composite Measure Pain Scale (CMPS-Feline) was used to assess postoperative pain in all patients at 2, 12, and 24 h after surgery [37,38]. Assessment was performed by three observers unaware of the analgesic treatment. This questionnaire consists of 7 behavioral categories (items) assessing spontaneous behavior, posture, and activity, attention to the wound or painful area, ear position, response to stroking, response to touch, and general impression of the cat in the cage. The items are numbered in ascending order of pain intensity. The total score for the CMPS-Feline was 20. If the pain score was >5/20, rescue analgesia was administered, consisting of a fentanyl bolus (2 µg/kg). Subjects requiring rescue analgesia were monitored for signs of pain but were excluded from the study.

### 2.3. Statistical Analysis

Sample size calculation was performed to determine the number of cats needed for this study. Sample size was calculated with the G*Power 3.1 Software (Heinrich-Heine-Universitat Dusseldorf, Düsseldorf, Germany). Accepting an effect size (f) of 0.45, a significance level (α) of 0.05, a power (1-β) of 0.85 with Anova fixed effects, omnibus, one-way test. Statistical analysis of the data set was performed using SPSS 15.0 (IBM Company, Milano, Italy). Normality of the data was tested using the Shapiro–Wilk test. Isoflurane requirement, and clinical parameters (HR, RR, SAP, MAP, DAP, EtCO_2_, SpO_2_) were analyzed using a two-way repeated measures ANOVA. Bonferroni post hoc pairwise comparison test between least squares means was used if statistical differences were present. Values corrected by SPSS were reported as mean ± standard deviation (SD). Scores relating to responses to noxious intraoperative stimulation and postoperative pain scores CMPS-Feline were also compared within and between groups using a Friedman test. As they are categorical data, median and range were computed. To measure the level of agreement between observers the Kendall’s coefficient of concordance W was calculated. Values *p* < 0.05 were considered statistically significant.

## 3. Results

No significant differences in age and weight were found between the cats enrolled, 20 cats in each group were necessary to recognize a statistically significant difference, and actual power was 0.86. Anesthesia and the postoperative period were uneventful, and all cats completed the study. Interobserver agreement was high (W = 1). No surgical complication was reported. The dose of alfaxalone used to induce anesthesia was not statistically different between groups and was administered according to patient requirements, ranging from 2.7 to 3 mg/kg. The anesthesia time was not statistically different among treatment groups (40 ± 3 min (mean ± SD)). Within the FT group, compared to T1, Fe’ISO was statistically lower at all time points, whereas within the F and T groups the significant reduction occurred at only a few time points. Among the groups, Fe’ISO showed a significant reduction in FT Group compared to F Group (*p* < 0.001) and T Group (*p* < 0.001), from T2 to T7. No difference among F Group and T Group (*p* > 0.05) was found (Table 1).

Table 2 shows the clinical parameters at the intraoperative time. Within all three groups, HR values compared to T1 were statistically lower at some time points (*p* < 0.001). The comparison of F and T Groups with the FT Group showed significantly higher HR values at some time points. No significant difference between the F Group and T Group was observed. In all groups, each cat breathed spontaneously. The RR values compared to T1 were lower at all time points in the T group (*p* < 0.001) and only at some time points in the FT group (*p* < 0.05). In the F Group an increase in breaths per minute compared to T1 was observed at some time point. At some time points, the F group showed higher RR values among the FT group (*p* < 0.05), while the T group showed lower RR values among the FT group (*p* < 0.05). The T Group showed lower RR values than among the F Group (*p* < 0.001). End-tidal CO_2_ values varied significantly within each group. At some time point EtCO_2_ values compared to T1 were lower in the F and T Group while they were higher in the FT Group (*p* < 0.001). The comparison of EtCO_2_ values between F, T, and FT Groups showed significant differences in some time points (*p* < 0.001). SpO_2_ did not vary significantly between groups at any time point, with optimal values always maintained around 99% (Table 2). Arterial blood pressure values (SAP, DAP, MAP) over the anesthetic period showed a downward trend in the F and FT Groups while an upward trend was shown in the T group. The comparison among groups confirmed that F and T Groups had lower blood pressure values than the FT Group at the same time points (*p* < 0.000). In addition, the T Group had lower blood pressure values than the F Group (*p* < 0.001). During anesthesia, the body temperature decreased in all cats, lowering by 2 ± 2 degrees (mean ± SD). There was no significant difference in body temperature among the groups.

The intraoperative noxious stimulation response scale showed lower scores than the baseline in all groups (*p* < 0.001). The T Group had higher scores at T2 to T7 than the other two groups (*p* < 0.001). The scores obtained showed a change in the monitored parameters (HR, RR, SAP) that did not require rescue analgesia (Table 3).

Cats in group FT were extubated earlier (3 ± 0.7 min (mean ± SD)) after isoflurane discontinuation compared with those in Group F (7 ± 0.4 min) and Group T (5 ± 0.6 min). Spontaneous sternal recumbency after extubation and infusion stop was observed at 10 ± 0.8 min in Group F, 20 ± 0.2 min in Group T, and 15 ± 0.6 min in Group FT. The patients were transferred to the intensive care unit for recovery, which was quick and quiet.

Based on the postoperative pain assessment, significant differences were observed between the groups according to the CMPS-Feline pain scoring system (Table 4). The score in each group did not exceed the cut-off value, and rescue analgesia was not required. In Group F a score reduction was observed at 12 (*p* = 0.03) and 24 h (*p* = 0.000) after surgery. The FT Group showed a reduction in score at 24 h (*p* = 0.001). No difference was observed in the T Group. Significant differences were observed among the three groups at all time points, with lower scores in the FT Group (*p* = 0.000).

## 4. Discussion

The results of this study showed that infusion of low doses of fentanyl combined with tramadol was clinically superior to either fentanyl or tramadol alone at higher doses in reducing Fe’ISO in cats undergoing ovariectomy. The combination provided a reduction in end-tidal isoflurane fraction, hemodynamic stability, low intraoperative noxious stimulation response, and postoperative pain assessment scores. The use of a multimodal analgesia with an association of different drugs may represent a valid method to provide adequate analgesia, improve anesthesia, and reduce side effects [22,32,39,40,41]. The first objective of this study was to determine whether infusion of fentanyl (F Group) or tramadol (T Group) alone or in combination (FT Group) would result in a reduction in isoflurane requirement in cats undergoing ovariohysterectomy. The data obtained in the groups show that in the F and T groups, a significant reduction in the end-tidal fraction of isoflurane (Fe’ISO) was observed only in the final phases (T5/T6–T7), whereas in the FT group this reduction occurred already in the initial phases (T2). The decrease in concentration at T6 and T7 in group F could be due to the lower intensity of these stimuli compared to T3, T4, and T5. The combination of drugs also had a greater sparing effect (28%) than fentanyl (22%) or tramadol (26%) alone. The end-tidal fraction of isoflurane during traction of both ovarian pedicles in cats receiving fentanyl and tramadol together was not different from that recorded during skin incision and suturing, indicating a beneficial effect of the drug combination. Our results are consistent with previous studies [5,19,25,42] showing that opioids reduce inhalational anesthetic requirements in cats with a plasma concentration and MAC-dependent effect [1,11,12,14,16,43]. It has also been reported in cats that the use of a fentanyl patch (25 or 50 µg/h) can reduce the requirement for isoflurane [7], while the administration of oral tramadol (8.6 to 11.6 mg/kg) can reduce the MAC of sevoflurane [44]. The second objective of this study was to compare the analgesic efficacy of fentanyl and tramadol alone with the fentanyl/tramadol combination. Response to intraoperative noxious stimulation was assessed by recording clinical parameters, while postoperative analgesia was assessed by recording pain scores. The intravenous co-administration of fentanyl and tramadol resulted in an improved analgesic plan and contributed to a hemodynamically stable anesthesia. During the experimental period, heart rate values in each group were shown to be statistically significant but remained within the physiological range for anesthetized cats [31]. Maintenance of adequate respiratory function is a primary requirement for safe anesthesia. In this study, each cat breathed spontaneously during anesthesia. In cats treated with tramadol alone, the respiratory rate at skin incision was lower than at endotracheal intubation. However, as our ETCO_2_ values suggest that the decreased rate was accompanied by an increase in tidal volume, there was no significant change in CO_2_. Significant variations in intraoperative EtCO_2_ recordings were seen in all groups, with values remaining within the target range during anesthesia [31]. Hemodynamic responses showed significant differences during anesthesia, but no treatment was required. These changes appear to be of little clinical relevance as mean heart and respiratory rates, end-tidal CO_2_, and arterial pressure were within clinically acceptable limits and comparable to those observed in other studies [10,26,45,46]. In particular, blood pressure in the T group was lower than in the other two groups throughout the entire observation period. This result is consistent with other experimental studies in cats and other species that have shown statistically lower blood pressure in subjects treated with tramadol compared to those treated with other drugs [8,10,30]. Unfortunately, it was not possible to perform an invasive blood pressure measurement, which is considered the gold standard [19,46,47]. Before and during surgery, the cats were placed on a circulating blanket of warm water. A gradual decrease in temperature during anesthesia was observed in all groups. This finding may be related to the susceptibility of cats to rapid temperature loss under anesthesia, partly due to their high surface area to body mass ratio [31]. However, the body temperature never dropped below the hypothermia threshold of 36.7 °C [45]. Intraoperative application of a cumulative noxious stimulation response scale showed that all three protocols had low noxious stimulation response scores, probably due to a balanced anesthetic protocol. Cats receiving fentanyl in combination with tramadol were extubated earlier than those receiving each drug separately. Spontaneous sternal recumbency was achieved at intermediate times in the FT group compared with the other two groups. As observed by other authors, this may be related to a faster washout of isoflurane in the FT group due to a lower requirement for isoflurane during anesthesia [10,23].

Some authors reported that a multimodal analgesia protocol includes a full µ-opioid receptor agonist, such as fentanyl, which provides dose-dependent analgesia and is preferred for moderate to severe pain, as well as an adjuvant analgesic, such as tramadol, which is administered for the treatment of severe acute pain and prevention of persistent postoperative pain [4]. Previous studies investigated the combination of tramadol with different analgesics to evaluate its antinociceptive effect as part of a multimodal analgesia plan in cats undergoing surgery [3,21,48,49,50], but no clinical studies have evaluated the combination of fentanyl and tramadol in an analgesic protocol for feline patients.

However, it is important to note that the pharmacokinetic properties of tramadol do not make it an ideal drug for CRI and that our study used only short-term administration to avoid complications due to accumulation (long-term administration). The dose of fentanyl used in this study in a multimodal regimen including dexmedetomidine (5 µg/kg) was chosen according to the clinical dose suggested for pain management in anesthetized cats [16,45,51,52,53,54]. In cats, tramadol has only weak µ-opioid activity and its active metabolite is produced and eliminated more slowly than in dogs. Therefore, tramadol should be used in the cat at lower doses and with longer dosing intervals than in the dog, as previously reported [22,24,25]. Tramadol has also been reported to produce analgesia by inhibiting the reuptake of norepinephrine and serotonin in the central nervous system [55]. There are no pharmacokinetic studies in the veterinary literature on the use of tramadol for CRI in cats. Previous studies investigated its effects on inductive drug requirements and cardiorespiratory variables in anesthetized cats [16,26,56] and dogs [57]. Although it has been reported that tramadol is eliminated more slowly in cats than in dogs [22], further studies are needed to investigate the trend of plasma tramadol concentrations in cats. Some authors agree on the preference of slow infusion of opioids alone or in combination with the use of boluses [19,58,59]. The combination of slowly infused fentanyl and tramadol can also be used to treat pain in conscious cats, providing continuous analgesia and avoiding the peaks, depressions, and breakthrough pain associated with intermittent boluses [19,24]. However, the inclusion of a bolus allows the plasma concentration of the drug to be increased, rather than waiting 4–5 half-lives to reach steady state. In our study with both tramadol (elimination half-life ~2 h) and fentanyl (elimination half-life ~2.5 h), the surgical procedure would be completed before reaching steady state. There are some limitations to this study. First, the use of oscillometric pressure, which is less accurate than direct pressure. The monitor used in this study was validated for oscillometric blood pressure measurement in anesthetized dogs according to the American College of Veterinary Internal Medicine (ACVIM) consensus criteria [60,61,62]. It would be interesting to investigate further to validate this multiparametric monitor for non-invasive oscillometric blood pressure measurement in anesthetized cats. Furthermore, the lack of a specific method to ensure the lowest concentration of isoflurane and the lack of accurate pharmacokinetic analysis to investigate the plasma concentration of intravenously administered opioids are other limitations of the study. Further studies may be required to determine the minimum alveolar concentration (MAC) in cats anesthetized with the protocol used in this study and to analyze plasma drug concentrations to confirm our results and justify the doses used.

## 5. Conclusions

The results of this study suggest that the anesthetic-sparing effect of the combination of fentanyl and tramadol infusions may allow a reduction in the required dose of isoflurane, thereby helping to reduce adverse effects on hemodynamic stability. All three treatments provided satisfactory intraoperative antinociceptive effect and postoperative analgesia without adverse effects in cats undergoing ovariohysterectomy. However, the combination of fentanyl and tramadol administered intravenously at the doses used in this study provided a better effect than the same drugs used alone. Therefore, the combination of fentanyl and tramadol could be proposed as a viable alternative anesthetic protocol in cats undergoing ovariohysterectomy.

## Figures and Tables

**Table 1:** End-tidal fraction of isoflurane (Fe’ISO) and its significance. Values are expressed as mean ± SD. Significance between groups: ° F Group vs. FT Group; ^♣^ T Group vs. FT group (*p* < 0.05). * Significant differences within group (*p* < 0.05).

	T1	T2	T3	T4	T5	T6	T7
F Group	1.8 ± 0.1	1.8 ± 0.2 °	1.7 ± 0.2 °	1.7 ± 0.2 °	1.7 ± 0.2 °	1.5 ± 0.2 *°	1.4 ± 0.2 *
T Group	1.9 ± 0.2	1.7 ± 0.2 ^♣^	1.8 ± 0.2 ^♣^	1.7 ± 0.2 ^♣^	1.6 ± 0.2 *^♣^	1.5 ± 0.1 *^♣^	1.4 ± 0.2 *
FT Group	1.8 ± 0.4	1.4 ± 0.2 *	1.3 ± 0.1 *	1.3 ± 0.3 *	1.3 ± 0.1 *	1.3 ± 0.2 *	1.3 ± 0.3 *

T1: Endotracheal intubation as basal values, T2: skin incision, T3: traction of the right ovarian pedicle, T4: traction of the left ovarian pedicle, T5: ligation of the uterine body, T6: beginning of the suture of the peritoneum, and T7: skin suturing.

**Table 2:** Intraoperative variables expressed as mean ± SD of HR, RR, SAP, MAP, DAP, EtCO_2_, SpO_2_, and T in cats treated with fentanyl (F Group), tramadol (T Group), and fentanyl/tramadol association (FT Group). * Significant differences within groups compared to T1 (*p* < 0.01). ^†^ Statistical difference Group F vs. Group FT. ^∞^ Statistical difference F Group vs. T Group. ^◊^ Statistical difference Group T vs. Group FT, at the same time point (*p* ˂ 0.05).

Variables	Groups	T1	T2	T3	T4	T5	T6	T7
HR (beats/minute)	F (192 ± 8)	165 ± 7	163 ± 7	163 ± 7	164 ± 6	161 ± 5 *	161 ± 6 *	160 ± 6 *
	T (187 ± 6)	167 ± 7	165 ± 8	166 ± 7	167 ± 6	167 ± 7	166 ± 5 *	159 ± 6 *
	FT (188 ± 9)	165 ± 9	164 ± 7	163 ± 6	163 ± 5	160 ± 5 *^◊^	148 ± 7 *^†◊^	146 ± 8 *^†◊^
RR (breaths/minute)	F (47 ± 9)	18 ± 2	21 ± 0 ^∞^*	20 ± 1 ^∞^	20 ± 1 ^∞^	21 ± 1 *^∞^	20 ± 0 ^∞^	19 ± 1 ^∞^
	T (46 ± 6)	20 ± 5	15 ± 4 *	16 ± 4 *	11 ± 2 *	12 ± 3 *	11 ± 3 *	12 ± 3 *
	FT (44 ± 5)	18 ± 6	19 ± 5	20 ± 5	16 ± 4 ^◊^	17 ± 4 ^†◊^	15 ± 3 *^†^	15 ± 3 *^†^
SAP (mmHg)	F (133 ± 8)	125 ± 13 ^∞^	120 ± 14 ^∞^	121 ± 14 ^∞^	115 ± 5 *	108 ± 7 *	99 ± 7 *	102 ± 7 *
	T (127 ± 9)	91 ± 5	92 ± 5	100 ± 5 *	97 ± 3 *	101 ± 5 *	107 ± 7 *	104 ± 7 *
	FT (135 ± 8)	130 ± 13 ^†◊^	115 ± 8 *^◊^	119 ± 8 *^◊^	111 ± 9 *^†◊^	116 ± 7 *^†◊^	113 ± 7 *^†◊^	125 ± 14 *^†◊^
MAP (mmHg)	F (102 ± 7)	90 ± 4 ^∞^	90 ± 4 ^∞^	92 ± 5 ^∞^	86 ± 6 *^∞^	78 ± 6 *^∞^	77 ± 6 *	67 ± 3 *
	T (100 ± 7)	66 ± 5	65 ± 4	65 ± 4	66 ± 5	68 ± 4	72 ± 4 *	71 ± 3 *
	FT (104 ± 9)	108 ± 8 ^†◊^	84 ± 4 *^†◊^	99 ± 9 *^†◊^	95 ± 8 *^†◊^	87 ± 5 *^†◊^	85 ± 3 *^†◊^	96 ± 3 *^†◊^
DAP (mmHg)	F (75 ± 6)	71 ± 6 ^∞^	72 ± 6 *^∞^	71 ± 6 *^∞^	69 ± 5 *^∞^	66 ± 3 *^∞^	64 ± 6 *^∞^	51 ± 7 *
	T (72 ± 9)	51 ± 7	46 ± 6 *	46 ± 6 *	52 ± 7	51 ± 7	53 ± 6	51 ± 8
	FT (83 ± 7)	82 ± 4 ^†◊^	69 ± 5 *^◊^	74 ± 5 *^◊^	77 ± 5 ^†◊^	69 ± 5 *^†◊^	67 ± 5 *^◊^	67 ± 2 *^†◊^
EtCO_2_ (mmHg)	F	37 ± 4 ^∞^	29 ± 4 *	29 ± 4 *	32 ± 3	29 ± 4 *^∞^	28 ± 4 *^∞^	31 ± 5 *^∞^
	T	35 ± 3	31 ± 3 *	32 ± 3 *	35 ± 4	36 ± 4	36 ± 5	38 ± 4
	FT	31 ± 3 ^†◊^	33 ± 3 ^†^	34 ± 3 *^†^	37 ± 4 *^†^	32 ± 3 ^◊^	36 ± 4 *^†^	34 ± 4 *^†◊^
SpO_2_ (%)	F	98 ± 1	99 ± 0	98 ± 2	96 ± 0	98 ± 2	99 ± 1	98 ± 2
	T	99 ± 1	99 ± 0	100 ± 0	100 ± 0	99 ± 0	100 ± 0	100 ± 0
	FT	99 ± 0	98 ± 2	99 ± 1	99 ± 1	99 ± 0	100 ± 0	100 ± 0
Body Temperature °C	F	38.6 ± 2	38.1 ± 3	37.7 ± 3	37.5 ± 2	37.4 ± 4	37.1 ± 3	37.6 ± 4
	T	38.5 ± 6	38.4 ± 4	38 ± 4	37.7 ± 2	37.3 ± 3	37.2 ± 4	37.5 ± 5
	FT	38.5 ± 5	38.1 ± 5	37.8 ± 3	37.5 ± 4	37.2 ± 5	37.3 ± 3	36.9 ± 2

In parentheses after the group identifier in the Group column: before premedication; T1: endotracheal intubation, T2: skin incision, T3: traction of the right ovarian pedicle, T4: traction of the left ovarian pedicle, T5: ligation of the uterine body, T6: beginning of the suture of the peritoneum, and T7: skin suturing.

**Table 3:** Cumulative intraoperative score for responses to noxious stimulations (percentage variations of HR, RR, and SAP compared with premedication values) in each group. Values, compared with T1 (endotracheal intubation), are expressed as median and range. ^♣^ Statistical differences between F Group and T Group vs. FT group (*p* < 0.001); ^∞^ Statistical difference F Group vs. T Group (*p* < 0.001); * Statistical differences within-group (*p* ˂ 0.05).

	T2	T3	T4	T5	T6	T7
F Group	1 ^∞^	0 *^∞♣^	0 *^∞^	1 ^∞^	0 ^∞♣^	0 ^∞^
	(0/1)	(0/0)	(0/0)	(0/1)	(0/1)	(0/1)
T Group	1 ^♣^	1 ^♣^	0 ^♣^	1 ^♣^	1 ^♣^	0 ^♣^
	(1/2)	(1/2)	(0/1)	(1/2)	(1/2)	(0/1)
FT Group	1	1	0	0	0 *	0 *
	(0/1)	(0/1)	(0/1)	(0/1)	(0/0)	(0/0)

**Table 4:** Glasgow Feline Composite Measure Pain Scale (CMPS-Feline). Values at 2, 12, and 24 h are expressed as median and range. * Significant within-group differences (*p* ˂ 0.05). ^♣^ Significant differences between groups (*p* < 0.000).

	2 h	12 h	24 h
F group	4.8 (3/5) ^♣^	4.5 (3/5) *^♣^	4.1 (2/5) *^♣^
T group	3.4 (0/5) ^♣^	3.7 (0/5) ^♣^	3.4 (0/5) ^♣^
F/T group	1.6 (0/3) ^♣^	1.4 (0/3) ^♣^	1 (0/2) *^♣^
	*p* = 0.000	*p* = 0.000	*p* = 0.000

## Data Availability

The datasets analyzed during the current study are available from the corresponding author on reasonable request.
